# Sociodemographic associations with uptake of novel therapies for acute myeloid leukemia

**DOI:** 10.1038/s41408-023-00964-x

**Published:** 2023-12-21

**Authors:** Andrew Hantel, Colin Cernik, Hajime Uno, Thomas P. Walsh, Gregory S. Calip, Daniel J. DeAngelo, Christopher S. Lathan, Gregory A. Abel

**Affiliations:** 1 Department of Medical Oncology, Dana-Farber Cancer Institute, Boston, MA USA; 2 Harvard Medical School, Boston, MA USA; 3 HMS Center for Bioethics, Boston, MA USA; 4 Flatiron Health, New York, NY USA; 5 University of Southern California, Los Angeles, CA USA

**Keywords:** Health services, Drug therapy

## Abstract

Inequitable uptake of novel therapies (NT) in non-cancer settings are known for patients with lower socioeconomic status (SES), People of Color (POC), and older adults. NT uptake equity in acute myeloid leukemia (AML) is not well known. We performed a retrospective cohort study (1/2014-8/2022) of the United States nationwide Flatiron Health^TM^ electronic health record-derived, de-identified database. We estimated sociodemographic associations with AML NT receipt using incidence rate ratios (IRR). Odds ratios (OR) assessed differences in venetoclax (the most common NT) receipt at community sites and between site characteristics and NT adoption. Of 8081 patients (139 sites), 3102 (38%) received a NT. NT use increased annually (IRR 1.14, 95% confidence interval [1.07, 1.22]). NT receipt was similar between Non-Hispanic-Whites and POC (IRR 1.03, [0.91, 1.17]) and as age increased (IRR 1.02 [0.97, 1.07]). At community sites, Non-Hispanic-Whites were less likely to receive venetoclax (OR 0.77 [0.66, 0.91]); older age (OR 1.05 [1.04, 1.05]) and higher area-level SES were associated with venetoclax receipt (OR 1.23 [1.05, 1.43]). Early NT adopting sites had more prescribing physicians (OR 1.25 [1.13, 1.43]) and higher SES strata patients (OR 2.81 [1.08, 7.66]). Inequities in AML NT uptake were seen by SES; for venetoclax, differential uptake reflects its label indication for older adults and those with comorbidities.

## Introduction

Since 2015, eight new therapeutics have been approved by the United States Food and Drug Administration (FDA) to treat acute myeloid leukemia (AML), and 5-year survival has surpassed 30% [1]. Despite these advances, outcome gains have not been equitably realized among the 64,000 Americans living with AML. Compared with non-Hispanic White (NH-White) individuals, NH-Black and Hispanic individuals with AML have 27% and 13% higher rates of death, respectively [2–4]. Survival in older adults also remains poor, with 5-year survival stagnant below 10% [3, 5], and neighborhood SES correlates with survival [6]. Multiple reasons have been offered to explain these sociodemographic differences including pathophysiologic factors that manifest in poor prognosis disease, patient factors such as increased treatment-related toxicity, and societal issues such as barriers to specialized care and structural racism [7–9]. Still, these issues have not sufficiently explained the sociodemographic differences seen, leaving an incomplete understanding of how outcomes disparities are perpetuated [2–4, 7, 8].

One aspect of AML treatment that has not been sufficiently investigated is how novel therapies diffuse into practice after FDA approval. Decreased and delayed uptake of high-cost, high-intensity drugs have been reported among racialized minorities and elderly populations after novel treatment approvals in non-cancer specialties [10, 11]. Some data suggest such delayed uptake extends to other blood cancers, such as multiple myeloma [12]. Data on novel therapy diffusion for AML are few. Given the number of recent FDA approvals, we sought to assess if there are sociodemographic inequities in the diffusion of novel therapies for AML treatment. Given the underlying inequities in AML outcomes, we hypothesized that older adults, people of color (POC), and those residing in areas with lower SES would be less likely to receive novel therapies during the years just following approval.

## Methods

### Study design and cohort

We performed a retrospective cohort study of adults diagnosed with AML from 1/2014-8/2022 using the Flatiron Health^TM^ (FH) electronic health record (EHR)-derived database, a longitudinal database comprising de-identified, patient-level, structured and unstructured data curated via technology-enabled abstraction from ~280 academic and community cancer clinics (800 sites of care) across the United States [13, 14]. It is not claims-based but instead uses data abstracted from the EHR. While the FH database has similar demographic and geographic distributions to other national databases including the Surveillance, Epidemiology and End Results Program and National Program of Cancer Registries, ~75% of patients in the FH database are treated in community practices, and it has greater representation of the American South.

Our primary objective was to characterize sociodemographic associations with novel therapy use, with a novel therapy defined as a drug that received FDA approval during the study period. These were glasdegib, venetoclax, ivosidenib, midostaurin, gemtuzumab ozogamicin, gilteritinib, enasidenib, and CPX-351. Exploratory objectives were to determine sociodemographic associations with venetoclax use (the most commonly prescribed novel therapy) and site-level characteristics associations with early novel therapy adoption. The study received approval from the Dana-Farber/Harvard Cancer Center Office for Human Research Studies. Restrictions apply to the availability of these data, which were used under license from FH. The data that support the findings of this study have been originated by FH, Inc. They are de-identified and subject to obligations to prevent re-identification and protect patient confidentiality. Requests for data sharing by license or by permission for the specific purpose of replicating results in this manuscript can be submitted to dataaccess@flatiron.com.

### Measures

Sociodemographic characteristics analyzed in the primary analyses included patient age, sex, and race-ethnicity, time from FDA approval to treatment event (defined below), practice type (academic or community), practice ID, and physician ID. Exposure time, defined as days between diagnosis and death or last follow up, was used as an offset variable. US population-weighted quantiles of an area-level measure of SES based on the Yost Index was available for patients treated at community sites of care [15]. SES rank-grouped patients based on a factor analysis of area-level characteristics from the American Community Survey (2015–2019) at the census block group level, including median household income, rent, poverty, employment, and education. SES was analyzed as low (bottom two quantiles) versus medium/high (top-three quantiles). Exploratory analyses of community sites were performed including SES as a covariate. The outcome measure was a treatment event, defined as the start of a new therapeutic regimen excluding hydroxyurea or research-based regimens. Treatment events were determined by oncologist-defined, rule-based lines of therapy specific for AML from technology-enabled abstraction and EHR-documented treatment administration data, then dichotomously categorized as including or not including a novel therapy. Treatment events were included starting with first-line treatment following diagnosis of AML and all subsequent line-of-therapy initiations until the end of the study period, end of EHR activity, or all-cause death determined from a combination of data from structured and unstructured EHR, commercial sources and the Social Security Death Index [16].

In primary analyses, race-ethnicity was defined dichotomously as POC or non-Hispanic (NH)-White, with POC an aggregate of individuals listed as NH-Asian, -Black, and -Other races and Hispanic Black, White, and Other races. An aggregated measure was used as small numbers for multiple race-ethnic groups made disaggregated models unstable. These categorizes and aggregations were based on the mutually exclusive combinations of race and ethnicity used by the FDA and the American Medical Association’s (AMA) definition of POC [17, 18]. The data source had two variables that indicated Hispanic status: a Hispanic indicator variable and a Hispanic race variable category. Hispanic status was identified through the Hispanic indicator in 99.5% of cases, and in 3.2% of cases, Hispanic was listed as a race category. In the primary analysis the POC term included individuals with Hispanic status identified through either variable. A sensitivity analyses were performed for each outcome using the race variable only, comparing POC as Hispanic, Asian, Black, or Other to White.

To determine site-level characteristics associations with early novel therapy adoption, we characterized sites as “early adopting” if they were below the median time to novel therapy use across the cohort (empirically identified as 91 days after FDA approval). Transformation to site-level variables was as follows. Race-ethnicity was transformed to the proportion of patients at a site who were POC; age and SES were transformed to the median values for patients at the site. Other site-level characteristics included the number of physicians in the dataset treating patients with AML and the number of treatment events at the site.

### Statistical analyses

Baseline characteristics were reported descriptively; Chi-square or Wilcoxon-Rank-Sum tests examined differences between those who did or did not receive a novel therapy. Differences in novel therapy treatment events by race-ethnicity were assessed through mixed-effects Poisson regression with exposure time as an offset variable and with patient race-ethnicity, age, sex, and treatment site type as fixed effects and practice ID as a random effect [19]. An exploratory analysis of community sites was performed using a similar model with SES included as a fixed effect. Differences in use for the most frequently used novel therapy, venetoclax, were assessed at community sites using multinomial regression with the same variables and controlling for the competing risk of treatment with another novel therapy. Multivariable logistic regression was used to assess associations between treatment site characteristics and being an early adopting site. Due to multicollinearity between number of treating physicians, total treatment volume, and number of patients treated, only number of treating physicians was included in the model. Similarly, SES and site region was strongly collinear and, thus, only SES was included.

All p-values were two-sided, and the significance level was set to 0.05. No adjustment for multiplicity was applied. All analyses were performed using R version 4.2.1 (the CRAN project, www.cran.r-project.org) and RStudio (Posit Software, Boston, MA). Packages *lme4* (for the mixed-effects Poisson regression) and *nnet* (for the multinomial regression) were used.

## Results

### Cohort characteristics

Of 8081 patients in 139 sites of care, 3102 (38%) received at least one novel therapy. Cohort characteristics are shown in Table 1. Patients were predominately older (69; interquartile range [57, 77]), male (57%), NH-White (72%), and treated at a community practice (74%). Venetoclax was the most frequently observed novel therapy (72% of novel treatment events). Patients receiving novel therapy typically received one treatment event containing a novel agent (median: 1 [1, 1]); they had more overall treatment events than the total cohort (median: 3 [2, 4]; *p* < 0.001). Bivariate associations were seen between novel therapy use and SES grouping (*p* < 0.001) for POC (*p* = 0.04), older age (*p* < 0.001), and practice type (*p* < 0.001; Table 1); similar associations were seen when assessed at the treatment event-level (results not shown).

**Table 1:** Cohort characteristics.

	Overall Patients	Patients Receiving Novel Therapy	
	*N* = 8081^a^	*N* = 3408^a^	*p*-value^b^
Total Treatment Events			<0.001
Median (IQR)	2 (2, 4)	3 (2, 4)	
Total Novel Treatment Events			N/A
Median (IQR)		1 (1, 1)	
Novel Therapy			N/A
Venetoclax	2453 (30%)	2453 (72%)	
Midostaurin	441 (5.5%)	441 (13%)	
CPX-351	423 (5.2%)	423 (12%)	
Enasidenib	181 (2.2%)	181 (5.3%)	
GO	281 (3.5%)	281 (8.2%)	
Ivosidenib	105 (1.3%)	105 (3.1%)	
Glasdegib	27 (0.3%)	27 (0.8%)	
Gilteritinib	235 (2.9%)	235 (6.9%)	
Age			<0.001
Median (IQR)	69 (57, 77)	70 (61, 77)	
Sex			0.8
Female	3 480 (43%)	1473 (43%)	
Male	4 601 (57%)	1935 (57%)	
Race-Ethnicity			0.040
POC	2025 (28%)	888 (29%)	
NH-White	5228 (72%)	2153 (71%)	
Yost Index SES Tercile			<0.001
High	2234 (41%)	974 (43%)	
Medium	1987 (36%)	753 (33%)	
Low	1287 (23%)	548 (20%)	
Practice Type			<0.001
Academic	1978 (24%)	900 (26%)	
Community	5945 (74%)	2427 (71%)	
Academic and Community	158 (2.0%)	82 (2.4%)	

^a^n (%) or Median (IQR).

^b^Wilcoxon rank sum test; Pearson’s Chi-squared test.

*IQR* interquartile range, *GO* gemtuzumab ozogamicin, *POC* people of color, *SES* socioeconomic status, *NH* non-Hispanic.

### Novel therapy use

**Fig. 1:**
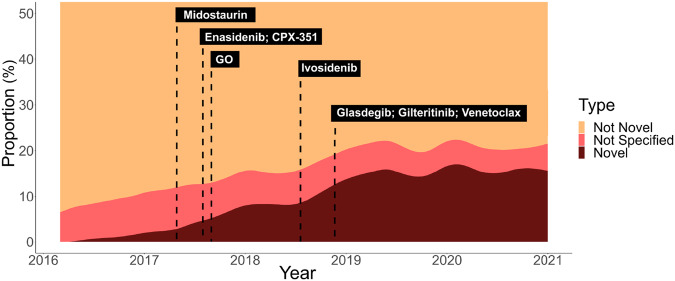
Novel therapy uptake. Stacked areas represent the proportion of all treatment events during each month of the study period that included novel therapies (brown), did not include novel therapies (tan), or were research therapies (red). Dashed lines represent dates of FDA approval for each novel therapy. % percentage, GO gemtuzumab ozogamicin.

Novel therapy prescription represented an increasing proportion of all treatment events observed in the study period, with its peak share plateauing at ~13% in 2020–2021 (Fig. 1). In mixed-effects Poisson regression analysis, use of a novel therapy increased every year after its FDA approval (IRR 1.14, [1.07, 1.22]). There was no difference in the likelihood of receiving a novel treatment for NH-White patients as compared to POC (IRR 1.03 [0.91, 1.17]). There were also no associations between older patient age (IRR 1.02 [0.97, 1.07]), male sex (IRR 0.97 [0.87, 1.08]) or treatment at a community site (IRR 1.14 [0.88, 1.48]). The full results of the mixed-effects Poisson regression model for the total population are shown in Table 2. In a sensitivity analysis with POC alternatively defined by only the race variable (Table S1), the likelihood of receiving a novel treatment was 20% percent higher for White patients as compared to POC (IRR 1.20 [1.05, 1.36]).

**Table 2:** Mixed-effects poisson regression (*N* = 6833).

Characteristic	IRR	95% CI	*p*-value
Race-Ethnicity			
POC	—	—	
NH-White	1.03	0.91, 1.17	0.6
Older Age^a^	1.02	0.97, 1.07	0.4
Sex			
Female	—	—	
Male	0.97	0.87, 1.08	0.5
Practice Type			
Academic	—	—	
Community	1.14	0.88, 1.48	0.3
Years After FDA Approval	1.14	1.07, 1.22	<0.001

^a^Ten-year increments.

*IRR* incidence rate ratio, *CI* confidence interval. *POC* people of color.

In an exploratory model among community sites only, results were concordant with the primary analysis, and no association between novel treatment and NH-White race was observed (IRR 1.02 [0.86, 1.21]; data not shown). When further adjusted for SES, there was no change in the estimate (IRR 1.09 [0.93, 1.27]), and there was no difference in the incidence rate ratio of novel therapy use between persons in the highest three SES quitiles as compared to those in the lowest two (IRR 1.06 [0.88, 1.27]; full results in Table S2). These results were concordant in a sensitivity analysis using the race variable-based definition of POC (results not shown).

### Venetoclax use at community practices

Of 3408 patients receiving novel therapy, 2453 (72%) received venetoclax. The patients receiving venetoclax community practices were older than the overall cohort (median: 75 [68, 80]). In a multinomial regression of patients treated at community sites, NH-White patients were less likely to receive venetoclax compared to POC (compared to non-novel therapy; OR 0.77 [0.66, 0.91]). Older age (OR 1.05 [1.04, 1.05]) and being from the highest three SES quintiles (as compared to the lowest two) were also associated with receiving venetoclax (OR 1.23 [1.05, 1.43]). Full model results are shown in Tables S3A, B. These results were concordant in a sensitivity analysis using the race variable only based definition of POC (results not shown).

### Characteristics of early adopting community practices

Of the 68 early adopting community practices, the mean and median number of novel therapy regimens used in the 91-day period that defined early adoption were 6 (standard deviation = 11) and 3 (interquartile range: 2, 5), respectively. Early adopting sites had significantly higher total treatment volume (combined novel and non-novel treatments; *p* < 0.001), lower average patient age (*p* = 0.009), mean patient SES in the highest three SES quintiles (*p* < 0.001) and a greater number of prescribing physicians (*p* < 0.001). Only 16% of early adopting centers, compared with 47% of late adopting centers, had median patient SES scores in the lowest two quintiles. There were no significant bivariate associations found between early adopting and non-adopting centers for the percentage of POC at the center (*p* = 0.3). Full center characteristics can be found in Table S4.

**Fig. 2:**
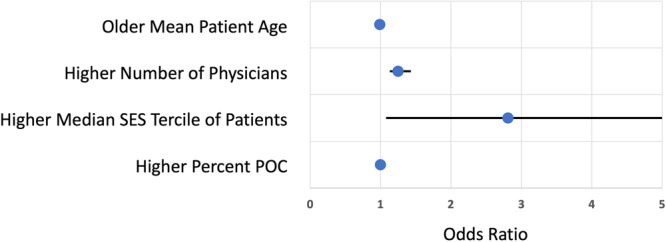
Site characteristics associated with early novel therapy adoption (N = 135 sites). Point estimates represent odds ratios of early novel therapy adoption associated with each covariate listed. Error bars represent 95% confidence intervals. The SES comparison is between sites with median patient SES in medium/high terciles vs low terciles. SES socioeconomic status, POC people of color.

In multivariable logistic regression (Fig. 2 and Table S5), conditionally independent associations with early adopting sites were the number of discrete prescribing physicians at a center (OR 1.25 [1.13, 1.43]) and SES (mean patient SES in quintiles 3–5 versus 1–2; OR 2.81 [1.08, 7.66]). There was no association between mean patient age at a site and early adopting status. These results were concordant in a sensitivity analysis that defined POC using only the race variable (results not shown).

## Discussion

In this large national retrospective cohort of patients with AML, older adults were as likely to receive recently FDA-approved treatments as younger adults, and they were more likely to receive venetoclax-based treatment. Novel therapy uptake by race and ethnicity was dependent on Hispanic ethnicity categorization, and results indicated that NH POC were less likely to receive novel therapies compared to White individuals. Among patients treated at community sites, there was no association between SES and any novel therapy use, though persons of higher SES were more likely to receive venetoclax-based treatment.

Patient-level findings were complemented by novel therapy use at the practice level, where sites caring for higher proportions of POC and older adults were as likely to be early adopters. Notably, sites with more physicians and those that treated patients with higher SES were likely to adopt novel therapies sooner. Contrary to our hypothesis, these data show that post-approval uptake inequities do not mirror those seen in AML clinical trial participation [20, 21], where research happens at academic sites and recruits more affluent, NH-White patients. They suggest that outcome inequities in AML are not clearly caused by differences in novel therapy use, though this may not be the case for persons from areas with lower SES. This aligns with a recent observational cohort study which found that a measure of structural racism (which included tract affluence) mediated a substantial proportion of observed inequities in AML survival by race and ethnicity [9].

Differences in uptake have been seen as contributors to inequitable care and outcomes across multiple medical specialties, but are less understood in AML [22]. Established inequities in innovative cancer care adoption have been characterized for conformal or intensity-modulated radiotherapy [23], targeted agents in myeloma [12], and digital mammography [24]. These differences follow common patterns of inequity in the United States, with historically marginalized groups and under-resourced communities less likely to obtain novel care. For adult AML, outcome disparities by race, ethnicity, and age have long been apparent, even in the decades when treatment options were stable and widely known [2–4]. Our data reveal that under-resourced communities appear to be at risk, but they do not strongly suggest inequities in novel treatment by race-ethnicity or age for these novel agents.

The lack of primary associations between POC and age with novel therapy use, coupled with their enhanced uptake of venetoclax-based treatment, suggests connections to therapy eligibility that may mask inequities. Venetoclax-based regimens are approved for adults 75 years or older or with co-morbidities that preclude the use of intensive regimens [25]. Several recent studies show that POC have increased comorbid burdens, which they are diagnosed with earlier in life [26, 27]. As data on comorbid status relevant to eligibility were not available, this unmeasured confounder and the popularity of outpatient oral therapies like venetoclax may be responsible for the higher level of uptake among POC. Still, the existence of novel therapies for these groups and having them used widely is undeniably good news for the AML community.

It is notable that there were novel therapy uptake inequities when POC was alternatively defined using only the race variable. As small numbers made modeling individual race-ethnic associations unstable, such aggregation was necessary (and performed according in alignment with the FDA and AMA) [18] but imperfect. This highlights the difficulty of assessing different dimensions of equity in rare cancer types, where even national samples are modestly sized. The inequity seen with this alternative definition suggests that POC identifying as Hispanic (i.e., only through the separate indicator variable) received novel therapies at a similar or higher rate compared to NH-Whites but that others aggregated as POC (e.g., NH Black and Asian persons) did not. This finding may reflect the geographic distribution of a dataset that is concentrated in the American South, where Hispanic populations are more prevalent and community sites may be more aligned with their care access needs.

Policy analysis has shown that high-cost and high-intensity medications have significant social system barriers to dissemination [28]. Despite this, the uptake of these expensive and intensive AML therapeutics was relatively broad and fast. Across community sites, the median time to first novel therapeutic prescription at the practice level was less than two months after FDA approval. This is substantially shorter than the 17-year period historically cited as the median time to adoption for new medical technologies or the two-year median identified for immune checkpoint inhibitors uptake [29, 30]. While these data do not directly assess the cause of this acceleration, adoption speed is associated with perceived benefits [22]. In this case, the previous lack of well-tolerated treatments for AML and the large increases in response and survival seen—especially for venetoclax—are likely responsible.

Strengths of this study include recency of data, national breadth of practices included, and community practice focus. Limitations include variable constraints placed by the data source, moderate sample size, and the retrospective nature of the study. Data source constraints included the lack of SES data for patients treated at academic centers, incomplete mutational data that limited our ability to constrict eligibility denominators for targeted agents, and other potentially confounding covariates that were unavailable (e.g., comorbid status). The sample size, while relatively large for an AML cohort, and retrospective nature of the cohort limited identification of covariates that could have been both important and significant. Indeed, future identification of potential diffusion inequities might include prospective patient- and physician-sourced data on behaviors, specific barriers to therapy prescription and/or receipt, and assessments of diffusion facilitators.

In conclusion, inequities in novel AML therapy uptake were not identified for POC or older adults, and these groups had increased uptake of venetoclax-based regimens. Novel therapy diffusion inequities in AML likely still exist for socioeconomically deprived communities seeking care at smaller practices, and future studies should prospectively assess diffusion and outcomes for these groups. Rational drug development continues to offer novel therapeutics at an impressive rate, and efforts to improve the diversity of participation in clinical trials are growing [31, 32]. Analogous assessments and interventions after FDA approval are needed to ensure equitable diffusion and realize outcome gains for all.

### Supplementary information

Supplemental Results

## Data Availability

The data that support the findings of this study have been originated by Flatiron Health, Inc. They are de-identified and subject to obligations to prevent re-identification and protect patient confidentiality. Requests for data sharing by license or by permission for the specific purpose of replicating results in this manuscript can be submitted to dataaccess@flatiron.com.
