# Supplementary material for: Sociodemographic associations with uptake of novel therapies for acute myeloid leukemia
Source: Blood Cancer J. 2023 Dec 21;13(1):192. doi: 10.1038/s41408-023-00964-x (PMC10733304; doi:10.1038/s41408-023-00964-x)
Supplement: Supplementary file 1 — Supplemental Results [file 41408_2023_964_MOESM1_ESM.docx]

**Supplemental Results**

**Table S1:** Mixed-Effects Poisson Regression (Race only) (N = 6833)

|  | IRR | 95% CI | p-value |
| --- | --- | --- | --- |
| Race |  |  |  |
| POC | — | — |  |
| White | 1.20 | 1.05, 1.36 | 0.007 |
| Older Age^1^ | 1.02 | 0.97, 1.06 | 0.5 |
| Sex |  |  |  |
| Female | — | — |  |
| Male | 1.02 | 0.92, 1.14 | 0.7 |
| Practice Type |  |  |  |
| Academic | — | — |  |
| Community | 1.08 | 0.77, 1.53 | 0.6 |
| Years After FDA Approval | 2.01 | 1.88, 2.14 | <0.001 |

^1^Ten-year increments

IRR: Incidence Rate Ratio; CI: Confidence Interval; POC: People of Color; FDA: Food and Drug Administration

**Table S2:** Community Subset Mixed-Effects Poisson Regression (N = 4730)

|  | **IRR** | **95% CI** | **p-value** |
| --- | --- | --- | --- |
| **Race-Ethnicity** |  |  |  |
| POC | — | — |  |
| NH-White | 1.09 | 0.93, 1.27 | 0.3 |
| **Older Age^1^** | 1.03 | 0.96, 1.09 | 0.4 |
| **Sex** |  |  |  |
| Female | — | — |  |
| Male | 1.07 | 0.94, 1.23 | 0.3 |
| **Mean SES Quintiles^2^** |  |  |  |
| Quintiles 1-2 | — | — |  |
| Quintiles 3-5 | 1.06 | 0.88, 1.27 | 0.6 |
| **Time After FDA Approval** | 1.63 | 1.51, 1.77 | <0.001 |

^1^Ten-year increments

^2^Lower quintiles indicate less affluent SES

IRR: Incidence Rate Ratio; CI: Confidence Interval; SES: socioeconomic status; POC: People of Color; NH: non-Hispanic; FDA: Food and Drug Administration

**Table S3A:** Venetoclax Multinomial Regression (N = 3687)

| ***Venetoclax (Comparator: Not Novel)*** | | | |
| --- | --- | --- | --- |
| **Sex** | **OR** | **95% CI** | **p-value** |
| Female | — | — |  |
| Male | 0.97 | 0.84, 1.12 | 0.7 |
| **Race-Ethnicity** |  |  |  |
| POC | — | — |  |
| NH-White | 0.77 | 0.66, 0.91 | 0.002 |
| **Older Age^1^** | 1.05 | 1.04, 1.05 | <0.001 |
| **SES Quintile^2^** |  |  |  |
| Quintiles 1-2 |  |  |  |
| Quintiles 3-5 | 1.23 | 1.05, 1.43 | 0.008 |

^1^Ten-year increments

^2^Lower quintiles indicate less affluent SES

OR: Odds Ratio; CI: Confidence Interval; SES: socioeconomic status; POC: People of Color

**Table S3B:** Venetoclax Multinomial Regression (Race only) (N = 3687)

| ***Venetoclax (Comparator: Not Novel)*** | | | |
| --- | --- | --- | --- |
| **Sex** | **OR** | **95% CI** | **p-value** |
| Female | — | — |  |
| Male | 0.94 | 0.80, 1.11 | 0.5 |
| **Race** |  |  |  |
| POC | — | — |  |
| White | 1.06 | 0.82, 1.37 | 0.7 |
| **Older Age^1^** | 1.05 | 1.04, 1.05 | <0.001 |
| **SES Quintile^2^** |  |  |  |
| Quintiles 1-2 |  |  |  |
| Quintiles 3-5 | 1.20 | 1.01, 1.42 | 0.04 |

^1^Ten-year increments

^2^Lower quintiles indicate less affluent SES

OR: Odds Ratio; CI: Confidence Interval; SES: socioeconomic status; POC: People of Color

**Table S4:** Characteristics of Early Adopting Sites

|  | **No**, N = 67*^1^* | **Yes**, N = 68*^1^* | **p-value***^2^* |  |
| --- | --- | --- | --- | --- |
| **Novel Treatment Events Per Patient at Site** |  |  | **<0.001** |  |
| Median (IQR) | 0 (0, 0) | 3 (2, 5) |  |  |
| **Total Treatment Events Per Patient at Site** |  |  | **<0.001** |  |
| Median (IQR) | 4 (2, 6) | 18 (19, 49) |  |  |
| **Site Region** |  |  | 0.7 |  |
| Midwest | 9 (17%) | 13 (19%) |  |  |
| Northeast | 9 (17%) | 14 (21%) |  |  |
| South | 27 (51%) | 27 (40%) |  |  |
| West | 8 (15%) | 13 (19%) |  |  |
| **Mean Patient Age at Site** |  |  | **0.009** |  |
| Median (IQR) | 68 (62, 72) | 63 (60, 68) |  |  |
| **Mean SES Quintile of Patients at Site** |  |  | **0.023** |  |
| Median (IQR) | 3 (2, 4) | 3 (3, 4) |  |  |
| **Percentage POC at Site** |  |  | 0.14 |  |
| Median (IQR) | 14 (0, 42) | 23 (9, 31) |  |  |
| **Number of Physicians at Site** |  |  | **<0.001** |  |
| Median (IQR) | 2 (1, 3) | 8 (5, 18) |  |  |
| **Number of Patients** |  |  | **<0.001** |  |
| Median (IQR) | 7 (4, 20) | 42 (22, 104) |  |  |
| **Majority Race-Ethnic Group** |  |  | 0.3 |  |
| POC | 11 (20%) | 9 (13%) |  |  |
| NH-White | 45 (80%) | 59 (87%) |  |  |
| *^1^* N (%) or Median (IQR) | | | | |
| *^2^* Wilcoxon rank sum test; Pearson's Chi-squared test | | | | |

IQR: interquartile range; SES: socioeconomic status; POC: People of Color; NH: non-Hispanic

**Table S5:** Logistic Regression for Early Adopting Sites (N = 135)

|  | **OR** | **95% CI** | **p-value** |
| --- | --- | --- | --- |
| **Older Mean Patient Age at Site** | 0.99 | 0.93, 1.06 | 0.9 |
| **Higher Number of Physicians at Site** | 1.25 | 1.13, 1.43 | <0.001 |
| **Mean SES of Patients at Site^1^** |  |  |  |
| Quintiles 1-2 | — | — |  |
| Quintiles 3-5 | 2.81 | 1.08, 7.66 | 0.038 |
| **Higher Percent POC at Site** | 1.00 | 0.98, 1.02 | >0.9 |

^1^Lower quintiles indicate less affluent SES

OR: Odds Ratio; CI: Confidence Interval; SES: socioeconomic status; POC: People of Color
